# Icariin attenuates diabetic cardiomyopathy by inhibiting NLRP3 inflammasome through SIRT3-mediated TFAM deacetylation

**DOI:** 10.3389/fphar.2026.1829772

**Published:** 2026-05-29

**Authors:** Min Sun, Hui Huang, Chao Wei, Yuan Xing, Bing Wu

**Affiliations:** 1 Department of Geriatrics, The 940th Hospital of Joint Logistics Support Force of Chinese People’s Liberation Army, Lanzhou, China; 2 The First Clinical Medical College of Gansu University of Traditional Chinese Medicine, Lanzhou, China; 3 Department of Neurology, The Second Medical Center, Chinese PLA General Hospital, Beijing, China; 4 Key Laboratory of Stem Cells and Gene Grug of Gansu Province, Department of Clinical Laboratory, The 940th Hospital of Joint Logistics Support Force of Chinese People’s Liberation Army, Lanzhou, China

**Keywords:** diabetic cardiomyopathy, icariin, mitochondrial DNA, NLRP3, oxidative stress, sirt3, TFAM

## Abstract

**Introduction:**

Icariin (ICA), a major flavonoid isolated from the traditional oriental herbal medicine Epimedium, exhibits various pharmacological activities, including anti-inflammatory and antioxidant properties. It has been demonstrated in previous studies that the NLRP3 inflammasome contributes to the progression of diabetic cardiomyopathy (DCM), and that ICA exerts a protective effect in DCM. However, the mechanism by which ICA may attenuate DCM by inhibiting the NLRP3 inflammasome still needs to be elucidated.

**Methods:**

The effects of ICA on the cardiac function and pathology of db/db mice were investigated. Levels of mitochondrial reactive oxygen species (mtROS), mitochondrial DNA (mtDNA), the NLRP3 inflammasome and pyroptosis were monitored in the hearts of DCM mice or H9c2 cardiomyoblasts induced by hyperglycaemia following ICA treatment. Furthermore, sirtuin 3 (SIRT3) activity and expression, as well as mitochondrial transcription factor A (TFAM) acetylation, were detected following ICA treatment.

**Results:**

We found that db/db mice treated with ICA exhibited significantly reduced cardiac hypertrophy and fibrosis, as well as improved cardiac function. ICA was associated with reduced mtROS, cytosolic mtDNA, NLRP3 inflammasome activation, and pyroptosis in the hearts of DCM mice or hyperglycaemia-induced H9c2 cardiomyoblasts, which may occur in a SIRT3-dependent manner. Ultimately, ICA-induced SIRT3 activation was associated with the deacetylation of TFAM. Discussion: These results suggested that ICA may attenuate DCM by inhibiting the NLRP3 inflammasome through a potential SIRT3-associated TFAM deacetylation pathway.

## 1 Introduction

Diabetes mellitus (DM) is a global public health problem and patients with DM often develop diabetic cardiomyopathy (DCM), which is characterized by the left ventricular hypertrophy, fibrosis, diastolic and systolic dysfunction, and cell signaling abnormalities. The development of DCM is a complex process driven by multiple factors such as inflammation, oxidative stress, myocardial cell death pathways, abnormal cardiac metabolism and neurohumoral activation (Ritchie and Abel, 2020).

Nucleotide-binding oligomerization domain (NOD)-like receptor protein-3 (NLRP3) is a key pattern recognition receptor (PRR) in the innate immune system. Previous research has suggested that chronic inflammation is one of the key drivers of DCM development, which can lead to cellular death by triggering the activation of NLRP3 inflammasome (Wang et al., 2024). Furthermore, NLRP3 inflammasome plays a crucial role in the progression of DCM and presents a promising therapeutic target (Guo et al., 2024). Previous studies have revealed that berberine can inhibit pyroptosis induced by NLRP3 inflammasome activation, thereby alleviating DCM (Zhong et al., 2024).

Mitochondrial DNA (mtDNA) is composed of multiple copies of circular DNA, approximately 16.6 kb in size, containing 37 genes that encode protein subunits crucial for oxidative phosphorylation (OXPHOS) (Lin et al., 2022). Emerging data suggests that a disruption in mitochondrial homeostasis leads to an overproduction of mitochondrial reactive oxygen species, which causes oxidative damage to mtDNA and its subsequent release into the cytoplasm. Cytosolic mtDNA can trigger the NLRP3 inflammasome, thereby exacerbating myocardial inflammation and pyroptosis in diabetic hearts (Yan et al., 2022). TFAM, a protein that binds to mtDNA, plays a vital role in preserving the stability of the mitochondrial genome. It manages the packaging, stability, and replication to ensure the mitochondrial genome’s abundance (Kang et al., 2018). In diabetic hearts, high glucose levels impaired PGC-1α-TFAM signaling, which is vital for mitochondrial biogenesis (Yang et al., 2024). Evogliptin prevented mitochondrial damage by activating PGC1a/NRF1/TFAM in the hearts of diabetic mice (Pham et al., 2023). Moreover, dysregulation and reduced levels of TFAM are crucial elements leading to defects in mtDNA packaging, causing mtDNA leakage (Song et al., 2024). In addition, Acetylation of TFAM at the K76 site influenced its import into mitochondria and reduced its ability to bind DNA, thus impairing mitochondrial biogenesis (Lv et al., 2022).

Icariin (ICA), representative active ingredient isolated from *Epimedium*, possesses antioxidant, anti-inflammatory and antifibrotic properties and has a great amount of attention in recent years (Zheng et al., 2024). ICA can increase Sesn2-induced mitophagy to inhibit NLRP3 inflammasome activation in diabetic nephropathy rats (Ding et al., 2022). Also, ICA can alleviate murine lupus nephritis, cadmium-induced renal injury, atopic dermatitis and ischaemic stroke through inhibiting NLRP3 inflammasome activation (Zheng et al., 2024; Su et al., 2018; Zhao et al., 2023; Liu et al., 2023). Moreover, ICA may have attenuated DCM development by preventing mitochondrial dysfunction through the Apelin/Sirt3 pathway (Ni et al., 2020). Our previous study proved that ICA protects cardiomyocytes against ischaemia-reperfusion injury by attenuating SIRT1-dependent mitochondrial oxidative damage (Wu et al., 2018).

However, whether ICA alleviates diabetic cardiomyopathy by suppressing the NLRP3 inflammasome through SIRT3-mediated deacetylation of TFAM is remains unknown. The goal of this study was to investigate if excessive mtROS and decreased TFAM activity might lead to mtDNA damage and its release into the cytoplasm, initiating NLRP3-mediated pyroptosis in cardiomyocytes, which could worsen the progression of diabetic cardiomyopathy. Additionally, this study was designed to assess whether ICA can lessen mtDNA damage and NLRP3-mediated pyroptosis by reducing mtROS and modulating SIRT3-mediated TFAM acetylation.

## 2 Materials and methods

### 2.1 Animal model and treatment

Seven-week-old male C57BL/6 J db/m (n = 6) and C57BL/6 J db/db mice (n = 18) were purchased from Taizhou Shuo Sen Biotechnology Co. Before the experiments, the mice were housed under specific pathogen-free (SPF) conditions, constant temperature (22 °C ± 2 °C) and humidity (50% ± 5%), provided with a uniform chow diet and water *ad libitum*, experiencing 12-h periods of light and darkness for at least 1 week to acclimate to their surroundings. Then the db/db mice were randomized to three groups: db/db group, ICA group and ICA + TYP group (n = 6/group). The db/db group received normal saline as control. ICA group and ICA + TYP group were administered Icariin (MedChem Express) orally at a dose of 30 mg/kg/day, as previously described (Zhang et al., 2021) for 12 weeks 3-TYP (Selleck Chemicals) at 5 mg/kg was administered to ICA + TYP group by intraperitoneal injection for consecutive 3 days after icariin treatment. At 24 h after the last 3-TYP injection, all the mice were anaesthetized, euthanized and samples of their hearts and serum were harvested for subsequent experimentation.

### 2.2 Cell culture and treatment

H9C2 cells were obtained from American Type Culture Collection (ATCC Manassas, VA, USA). The cell lines were maintained in Dulbecco’s modified Eagle’s medium (DMEM, Gibco, USA) supplemented with 10% fetal bovine serum (FBS, Biological Industries, Israel), 100 U/mL penicillin, and 100 μg/mL streptomycin, at 37 °C in a humidified atmosphere containing 5% CO_2_. H9C2 cells were randomly divided into two groups: a normal glucose (NG) group (5.5 mM glucose) and a high glucose (HG) group (33 mM glucose). The HG group was then divided into two subgroups: ICA subgroup (33 mM glucose +10 μM icariin), ICA + si-SIRT3 subgroup (33 mM glucose+10 μM icariin + SIRT3 siRNA intervention). The final concentration of SIRT3 siRNA was 50 nM, and the siRNA was transfected into H9C2 cells according to the manufacturer’s instructions, 6 h prior to icariin treatment. Cells were cultured in DMEM to reach approximately 90% confluence (48–72 h) before intervention, and each group was treated for 48 h.

### 2.3 siRNA transfection

Approximately 2 × 10^5^ H9C2 cells per well were grown in six-well plates and siRNA transfection was performed when the cell density reached 60% confluence. The siRNA was purchased from Santa Cruz Biotechnology (SIRT3: sc-61556; Control siRNA: sc-365175). Transfection was performed using Lipofectamine RNAiMAX reagent (Thermo Fisher Scientific; USA; Cat. No.:13,778), according to the manufacturer’s protocol. After transfection, the cells were incubated in a 37 °C, humidified 5%CO_2_ incubator for 48 h prior to subsequent experiments.

### 2.4 Isolation and detection of cytosolic mtDNA by qPCR

After lysing the heart tissue of DCM mice in cell lysis buffer (BioVision, Cat. No.: 1,067–100), all samples were centrifuged at 700 *g* at 4 °C for 10 min to separate out nuclei and intact cells. The supernatant was adjusted based on protein concentration (measured by BCA protein assay kit, Thermo Fisher Scientific). Subsequently, to isolate the cytosolic fraction, it was subjected to centrifugation at 10,000×g for 30 min at 4 °C. Subsequently, quantitative PCR (qPCR) was employed to quantify the levels of cytosolic mitochondrial DNA (mtDNA). Primers were designed according to the sequences encoding mtDNA, with the specific primer sequences detailed in Table 1 (Yan et al., 2022). The data were analyzed utilizing the 2^(−ΔΔCt)^ method and were normalized against β-actin.

**TABLE 1:** Primer sequences used for qRT-PCR.

Species	Gene	Forward	Reverse
Mouse	Dloop1	AAT​CTA​CCA​TCC​TCC​GTG​AAA​CC	TCA​GTT​TAG​CTA​CCC​CCA​AGT​TTA​A
	CytB	GCT​TTC​CAC​TTC​ATC​TTA​CCA​TTT​A	TGT​TGG​GTT​GTT​TGA​TCC​TG
	16s	CACTGCCTGCCCAGTGA	ATACCGCGGCCGTTAAA
	ND4	AACGGATCCACAGCCGTA	AGTCCTCGGGCCATGATT
	β-actin	GTG​ACG​TTG​ACA​TCC​GTA​AAG​A	GCC​GGA​CTC​ATC​GTA​CTC​C

### 2.5 Western blotting analysis and immunoprecipitation

The myocardial tissue and cells were treated with RIPA cell lysis buffer containing protease inhibitors (R0010, Solarbio Life Sciences). The protein concentration was determined using the BCA method (Solarbio Life Sciences, PC0020). The protein samples were then prepared by adjusting the concentration as required. Equal amounts of the samples were separated using 12% SDS-PAGE gels and transferred to PVDF membranes. The membranes were blocked with 5% skimmed milk at room temperature for 1 h, followed by an overnight incubation at 4 °C with the following primary antibodies: SIRT3 (1:1,000, Cell Signaling Technology); TFAM (1:800, Santa Cruz Biotechnology); NLRP3 (1:2000, Proteintech Group); ASC (1:1,000, ABclonal Technology); Caspase1 p20 (1:1,000, AdipoGen); pro-Caspase1 (1:1,000, MedChemExpress); GSDMD-N (1:2000, Immunoway); GSDMD (1:1,000, Affinity); IL-1β (1:2000, Proteintech); IL-18 (1:2000, Proteintech) and VDAC1 (1:500, Affinity) and β-Tubulin (1:50,000, Affinity). The membranes were then washed three times with TBST for 5 min each. After this, they were incubated with secondary antibodies at room temperature for 1 h. The results were visualized using chemiluminescence, observed and photographed using a gel imaging system, and finally quantified using ImageJ software. For immunoprecipitation analysis, lysates were mixed with the primary antibody. The immunocomplexes were then separated by SDS-PAGE and detected using a Western blot analysis.

### 2.6 Echocardiography

The DCM mice were anaesthetized with 12 μL/g body weight (BW) of 2.5% Avertin (Sigma-Aldrich), after which their cardiac function was determined using echocardiography (VisualSonics VeVo 770).

### 2.7 Histological analyses

Samples of left ventricular tissue were fixed in 4% paraformaldehyde for 48 h at room temperature and embedded in paraffin wax. The processing and staining of the tissue sections were conducted in accordance with the standard procedure, employing hematoxylin and eosin or Masson staining. Cardiac morphology was examined using a Nikon microscope (Japan).

### 2.8 WGA staining

The animal hearts were paraffin-embedded after fixation in 4% formaldehyde for 24 h and sectioned into 5 µm-thick slices. Cross-sectional areas of cardiomyocytes in the left ventricle (LV) were observed using fluorescein-conjugated wheat germ agglutinin (WGA; 5 μg/mL, AAT Bioquest) and evaluated by measuring the cross-sectional areas of individual myocytes using ImageJ software.

### 2.9 Isolation of cytoplasmic and mitochondrial fractions

Cells were collected through gentle scraping into the culture medium and subsequently washed with ice-cold phosphate-buffered saline (PBS, pH 7.4). mitochondrial fractions were isolated utilizing a mitochondria isolation kit (beyotime, cat. No.: C3601). The resulting pellet comprised the mitochondrial fraction, whereas the supernatant was retained as the cytosolic fraction

### 2.10 Quantification of mitochondrial reactive oxygen species (mtROS)

The quantification of mtROS levels was conducted utilizing MitoSOX™ Red mitochondrial superoxide indicators (Invitrogen, Cat. No.: M36008). According to the instructions outlined in the MitoSOX™ Red Reagent protocol, the reagent powder was dissolved in dimethyl sulfoxide (DMSO) to prepare a 5 μmol/L working solution. Post-treatment, the cells were rinsed with Phosphate Buffered Saline (PBS) and subsequently incubated with 1 mL of the working solution for 30 min at 37 °C under 5% CO_2_ conditions. Following incubation, the cells underwent three gentle washes with a warm buffer solution (Hank’s Balanced Salt Solution with Calcium and Magnesium). The cells were then examined using a confocal microscope (Leica, Germany).

### 2.11 Sirt3 deacetylation assay

The deacetylase activity of Sirt3 was assessed using a fluorometric assay kit (Cyclex, Nagoya, Japan), following the protocol provided by the manufacturer. In this assay, a reaction mixture was prepared comprising a fluorine-substrate peptide, lysyl-endopeptidase, NAD+, and Sirt3 assay buffer. The reaction was initiated by adding an enzyme sample free of protease inhibitors, after which the mixture was thoroughly mixed. Fluorescence intensity was measured with an excitation wavelength of 355 nm and an emission wavelength of 460 nm, while maintaining a constant reaction rate.

### 2.12 Statistical analysis

The results are presented as the mean ± SD. Multigroup data were analyzed using a t-test (for two groups) or one-way ANOVA with SPSS 19.0 software. Data were plotted using GraphPad Prism five software. A p-value of less than 0.05 was considered statistically significant.

## 3 Results

### 3.1 Escape of mtDNA into the cytosol and activation of the NLRP3 pathway in the hearts of DCM mice

**FIGURE 1:**
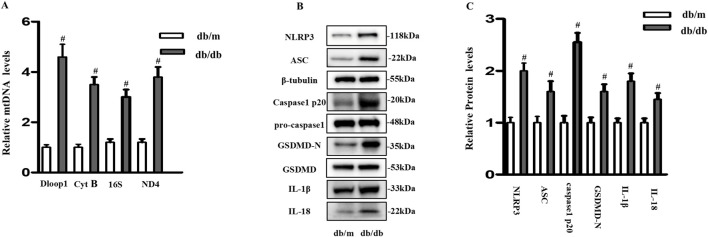
Cytosolic mtDNA levels and activation of the NLRP3 inflammasome and pyroptosis in the hearts of DCM mice. **(A)** mRNA levels of D-loop1, CytB, 16S, and ND4, determined by qRT-PCR. **(B,C)** Protein expression and quantitative analysis of NLRP3, ASC, caspase-1 (p20/pro-caspase-1), and GSDMD- N/GSDMD, as well as IL-1ẞ and IL-18 in mouse hearts, detected by Western blotting. Data are presented as the mean ± SE. ^#^P<0.05 vs. db/m group.

Cytoplasmic mitochondrial DNA (mtDNA) is recognized as a potent damage-associated molecular pattern (DAMP) in inflammatory diseases, particularly following oxidative damage due to the accumulation of mitochondrial reactive oxygen species (mtROS). In this study, we quantified cytosolic levels of mtDNA markers, including the displacement loop (Dloop1), cytochrome b (CytB), 16 S rDNA (16 S), and NADH dehydrogenase subunit 4 (ND4) in the cytosol and observed a significant upregulation of these markers in the cytosol of db/db mice compared to the db/m group (Figure 1A), which suggests the translocation of mtDNA into the cytosol. Subsequently, we examined the NLRP3 inflammasome and found that the NLRP3 signaling pathway was markedly activated in the hearts of db/db mice. This activation was accompanied by elevated protein levels of NLRP3, ASC, caspase-1 p20, GSDMD-N, as well as inflammatory cytokines (IL-1β and IL-18) in the hearts of db/db mice compared with db/m mice (Figures 1B,C). These findings suggest that the translocation of mtDNA into the cytosol may contribute to the activation of the NLRP3 signaling pathway, thereby potentially eliciting an inflammatory response in the hearts of DCM mice.

### 3.2 The administration of ICA was associated with a significant improvement in cardiac function in mice with DCM

**FIGURE 2:**
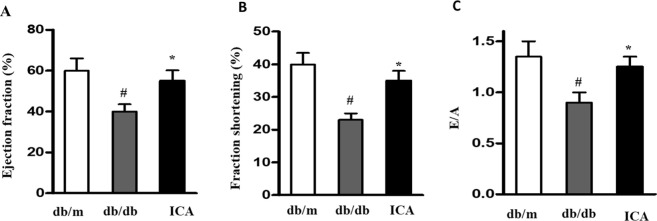
Effects of ICA on cardiac systolic and diastolic function in db/db mice. **(A)** Ejection fraction (EF%) .**(B)** fractional shortening (FS%). **(C)** Ratio of the velocities of early to late mitral flow (E/A). n = 6 in each group. ^#^P<0.05 vs db/m group, *P<0.05 vs db/db group.

Echocardiographic assessments were performed to evaluate the cardiac function of diabetic mice, focusing on *in vivo* left ventricular (LV) function. The echocardiographic data revealed that db/db mice exhibited significantly impaired in systolic and diastolic function compared to db/m mice. By contrast, db/db mice treated with ICA displayed a notable improvement in ejection fraction (EF), fractional shortening (FS), and the E/A ratio following a 12-week treatment period (Figures 2A–C).

### 3.3 ICA was associated with reduced cardiac hypertrophy and fibrosis in mice with DCM

**FIGURE 3:**
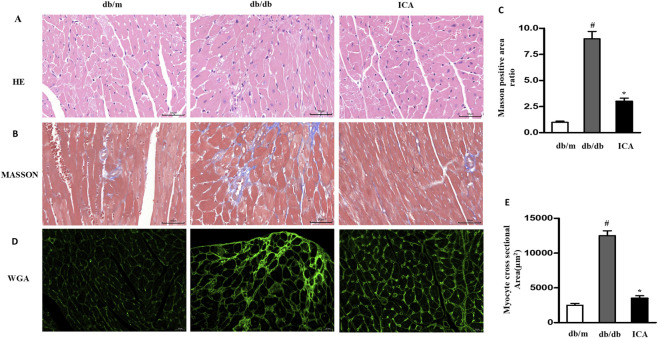
**(A–C)** Representative images of HE staining and Masson staining of heart sections in different groups. **(D,E)** Quantitative analysis of myocardial cross-sectional area (assessed by WGA staining) in each group. Magnification: x400. n = 6 per group. ^#^P<0.05 vs. db/m group; *P<0.05 vs. db/db group.

Hematoxylin and eosin (HE), wheat germ agglutinin (WGA), and Masson’s trichrome staining were used to assess the impact of ICA on diabetic cardiomyocyte hypertrophy and fibrosis. Histological observations showed that cardiomyocytes in db/m mice were distinctly striated and regularly organized, whereas those in db/db mice exhibited disorganized myocardial fibers and an increased myocyte cross-sectional area, as revealed by HE staining (Figure 3A) and WGA staining (Figures 3D,E). In addition, Masson’s trichrome staining showed enhanced myocardial interstitial fibrosis and collagen deposition in the hearts of db/db mice compared to db/m mice (Figures 3B,C). Notably, these pathological alterations were markedly attenuated in ICA-treated db/db mice. In conclusion, these results suggest that ICA treatment may effectively reduce cardiac hypertrophy and fibrosis.

### 3.4 ICA mitigates mtROS production in hyperglycemia-induced H9c2 cardiomyoblasts

**FIGURE 4:**
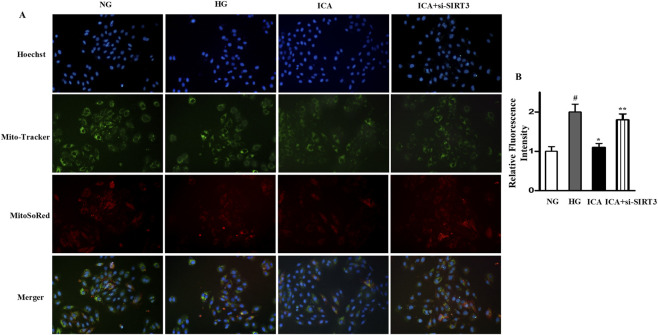
ICA is associated with alleviated mitochondrial oxidative stress under high glucose conditions in vitro, which may be mediated by SIRT3. **(A)** Confocal images showing mitochondrial reactive oxygen species (mtROS) (stained with MitoSOX) and mitochondrial network (stained with MitoTracker) in H9c2 cells. **(B)** Quantitative analysis of MitoSOX fluorescence intensity (mtROS level) and mitochondrial network integrity in H9c2 cells. Data are presented as the mean ± SE. P < 0.05 vs. NG group (normal glucose group); *P < 0.05 vs. HG group (high glucose group);**P<0.05 vs. ICA group (ICA treatment group).

Current evidence indicates that mitochondrial DNA (mtDNA) is vulnerable to damage by mitochondrial reactive oxygen species (mtROS). To evaluate the effect of ICA on mtROS production, confocal microscopy was employed to assess mtROS generation through staining with MitoSOX (red) and MitoTracker (green). Quantitative colocalization analysis demonstrated a significant increase in the bright red fluorescence of MitoSOX in the high glucose (HG)-induced group. Importantly, this increase was reversed following ICA treatment, suggesting that ICA may suppresses excessive mtROS generation in H9c2 cells exposed to HG. Conversely, this protective effect was markedly attenuated when SIRT3 knockdown using siRNA (Figures 4A,B).

### 3.5 ICA is associated with reduced cytosolic mtDNA leak, NLRP3 inflammasome activation and pyroptosis in the hearts of DCM mice in a SIRT3-dependent manner

To evaluate the effects of ICA on mtDNA damage and NLRP3-mediated pyroptosis, RT-qPCR and Western blotting were employed to quantify the levels of cytosolic mtDNA and the expression of key proteins associated with pyroptosis, specifically GSDMD and its cleaved form GSDMD-N, as well as the components of the NLRP3 inflammasome, including NLRP3, ASC, and caspase-1, in cardiac tissue. Additionally, transmission electron microscopy was utilized to identify pyroptotic vesicles in cultured H9c2 cardiomyocytes.

**FIGURE 5:**
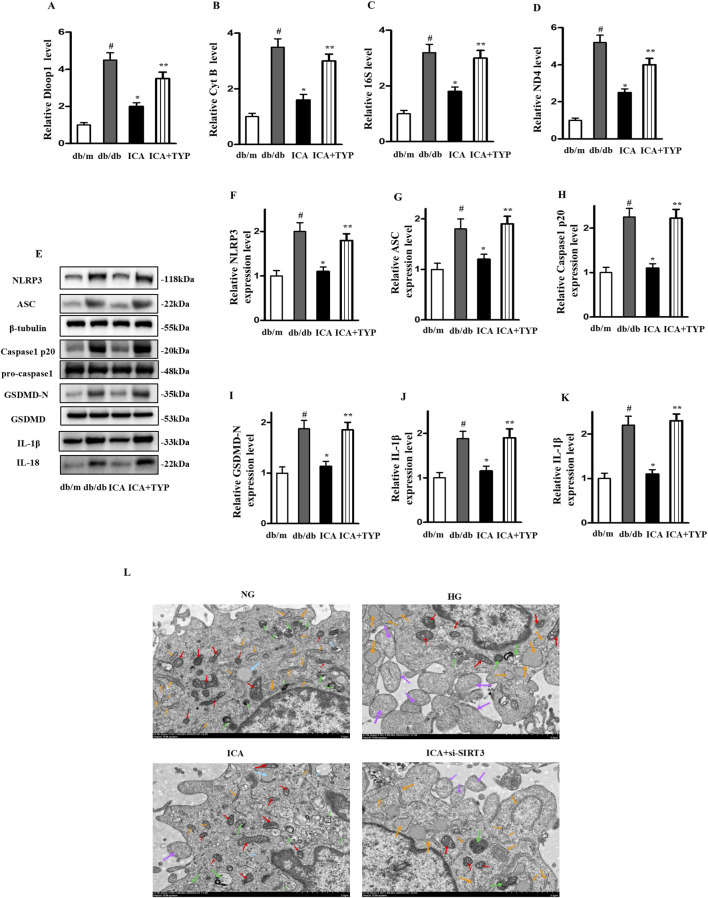
ICA is associated with reduced cytosolic mtDNA release, NLRP3 inflammasome activation, and pyroptosis in DCM mice, which may occur in a SIRT3-dependent manner. **(A–D)** mRNA levels of D-loopl, CytB, 16S, and ND4 were detected by qRT-PCR. **(E–K)** Protein expression and quantitative analysis of NLRP3, ASC, caspase-1 p20, GSDMD-N, IL-1ẞ, and IL-18 in mouse heart tissue were detected by Western blotting. **(L)** Representative TEM images of pyroptosis in H9c2 cells (original magnification: x7,000). The red arrow indicates the mitochondria; the orange arrow indicates the rough endoplasmic reticulum; the green arrow indicates the secondary lysosome; the purple arrow indicates the pyroptosis body. Data are presented as the mean ± SE. ^#^P<0.05 vs. db/m group; *P<0.05 vs. db/db group; **P<0.05 vs. ICA group.

RT-qPCR analysis demonstrated an upregulation in the levels of cytosolic D-Loop1, CytB, 16S, and ND4 genes in the cardiac tissues of the db/db group, compared with the db/m group. Notably, the administration of ICA significantly reduced the levels of these genes in diabetic cardiomyopathy (DCM) hearts (Figures 5A–D). Furthermore, Western blotting indicated a markedly elevation in the expression of NLRP3, ASC, caspase-1 p20, and GSDMD-N in the cardiac tissues of db/db mice relative to db/m mice. However, these elevated levels were significantly reduced following ICA treatment (Figures 5E–K). Transmission electron microscopy revealed that the outer mitochondrial membrane in the normal glucose (NG) group remained largely intact, with a medium-density mitochondrial matrix and an intact cell membrane. Conversely, in the high glucose (HG) group, the outer mitochondrial membrane and mitochondrial cristae were extensively lysed, the cell membrane exhibited partial loss of cell membrane integrity, and there was an increased presence of pyroptotic vesicles outside the cell membrane. Importantly, ICA treatment significantly preserved mitochondrial morphology and cell membrane integrity, while also reducing the number of pyroptotic vesicles (Figure 5L).

Interestingly, the protective effects of ICA on mtDNA leak, NLRP3 inflammasome activation, and pyroptosis were markedly attenuated when SIRT3 was inhibited by 3-TYP or knocked down by siRNA. These findings suggest that ICA may alleviate DCM by reducing cytosolic mtDNA leakage and NLRP3 inflammasome-mediated pyroptosis, which is likely dependent on SIRT3 activation.

### 3.6 ICA-induced SIRT3 activation is associated with TFAM deacetylation

**FIGURE 6:**
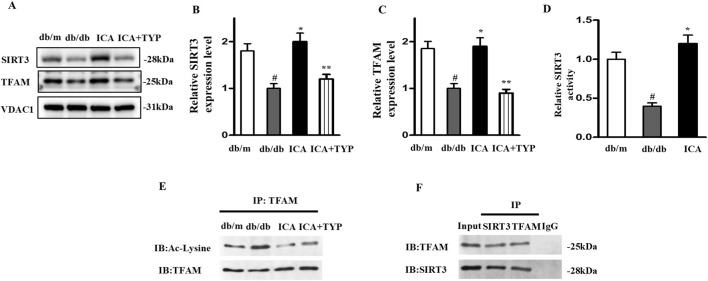
ICA-induced SIRT3 activation is associated with TFAM deacetylation. **(A–C)** Protein expression and quantitative analysis of SIRT3 and TFAM in mouse heart tissue determined by Western blotting. **(D)** SIRT3 activity in mouse heart tissue measured by ELISA assay. **(E)** Acetylation level of TFAM in mouse heart tissue examined by immunoprecipitation (IP). **(F)** Physical interaction between SIRT3 and TFAM in mouse heart tissue determined by co- immunoprecipitation (Co-IP).Data are presented as the mean ± SE.^#^P<0.05 vs. db/m group; *P<0.05 vs. db/db group; **P<0.05 vs. ICA group.

Western blotting and immunoprecipitation (IP) were performed to assess the impact of ICA on the expression and activity of SIRT3 and TFAM. The findings indicated a reduction in both the expression and activity of SIRT3 in diabetic mice, whereas ICA treatment was associated with a significant restoration of SIRT3 expression and activity (Figures 6A–D). In diabetic mice, TFAM expression was downregulated compared to db/m mice, while TFAM acetylation levels were markedly increased. Following ICA treatment was associated with a notable reduction in TFAM acetylation; however, this effect was attenuated by SIRT3 inhibition, as confirmed by immunoprecipitation in mouse heart tissue (Figure 6E). Co-immunoprecipitation (Co-IP) analysis suggested that ICA may potentially facilitates a physical interaction between SIRT3 and TFAM in mouse heart tissue (Figure 6F). These results suggest that ICA may activate SIRT3, which may subsequently mediates the deacetylation of TFAM.

## 4 Discussion

**FIGURE 7:**
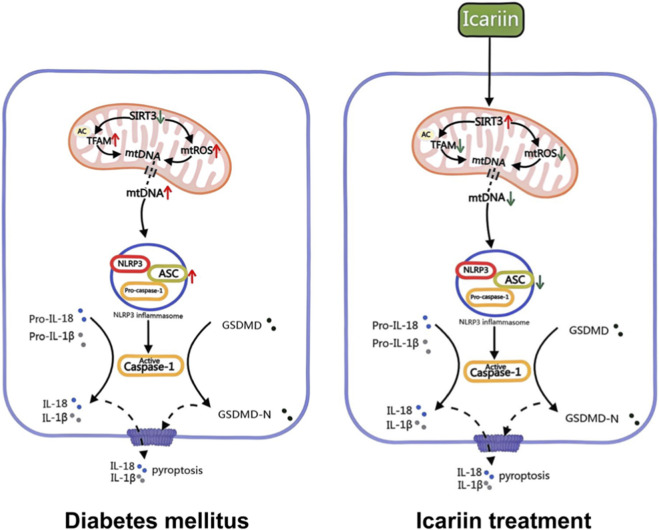
Schematic diagram illustrating the findings of this study. High glucose-induced mtROS accumulation is associated with mtDNA leakage, which may contribute to NLRP3 inflammasome activation in H9c2 cells, thereby promoting the release of proinflammatory cytokines and cardiomyocyte pyroptosis. ICA treatment is associated with enhanced SIRT3 activity, reduced mtROS generation, and decreased TFAM acetylation. These changes may be linked to suppressed mtDNA leakage, NLRP3 inflammasome activation, and pyroptosis, which could contribute to the protective effects of ICA against diabetic cardiomyopathy (DCM).

In the present investigation, our findings suggested that mitochondrial damage is associated with mitochondrial DNA (mtDNA) leakage and activation of the NLRP3 inflammasome pathway, which was associated with pyroptosis in the hearts of mice with diabetic cardiomyopathy (DCM), thus potentially exacerbating cardiac injury. Furthermore, ICA was found to be associated with enhanced SIRT3 activity and expression, reduced mitochondrial reactive oxygen species (mtROS) production, and improved TFAM expression and activity. These changes were associated with reduced cytosolic mtDNA accumulation, NLRP3 inflammasome activation, and pyroptosis in the hearts of DCM mice (Figure 7).

Mitochondrial dysfunction is one of the major factors contributing to the development of diabetic cardiomyopathy (Jubaidi et al., 2020). Increased mitochondrial fission, defective mitophagy, and disrupted mitochondrial biogenesis exacerbate mitochondrial dysfunction containing damaged mtDNA (Chang et al., 2022). The release of damaged mtDNA into the cytoplasm drives pyroptosis and proinflammatory responses in cardiomyocytes in a NLRP3 inflammasome-dependent manner (Yan et al., 2022). Consistent with previous study, we found that large amounts of mtDNA were present in the cytoplasm of diabetic cardiomyocytes. Cytosolic mtDNA acts as a signalling molecule that activates Toll-like receptor 9 (TLR9), the NLRP3 inflammasome and the cyclic GMP-AMP synthase (cGAS)-stimulator of interferon genes (STING) innate immune signalling pathways in response to foreign pathogens, and also induces the onset and progression of inflammatory diseases (Tao et al., 2024; Wu et al., 2021). In this article, we also found that the NLRP3 inflammasome and proinflammatory factors were elevated in the hearts of DCM mice at the same time. The NLRP3 inflammasome is a key sensor that mediates innate immune and inflammatory responses. Recent evidence has indicated that the NLRP3 inflammasome/pyroptosis pathway is strongly linked to DCM (Zhang et al., 2022), and modulating the NLRP3 inflammasome is an effective method of preventing and treating DCM (Sun and Ding., 2021).

Evidence has shown that ICA improves structural damage and myocardial fibrosis in rats with type 2 diabetes mellitus (T2DM) (Zhang et al., 2021). ICA also has preventive and therapeutic effects on diabetic cardiomyopathy by preventing mitochondrial dysfunction (Wu B et al., 2018). In the present study, we found that ICA was associated with reduced cardiac hypertrophy and fibrosis, as well as improved cardiac function in DCM mice. ICA possesses anti-inflammatory properties and has gained widespread attention in recent years. It might reduce the renal inflammatory response by suppressing the TLR4/NF-κB signalling pathway (Qi et al., 2021). Furthermore, ICA has been shown to inhibit NLRP3 inflammasome activation via the Keap1-Nrf2/HO-1 axis in rats with diabetic nephropathy (Ding et al., 2022). However, it remains to be established whether ICA treatment alleviates inflammation in DCM hearts by limiting NLRP3 inflammasome activation, as well as the underlying molecular mechanism. Our studies suggest for the first time that ICA may reduce NLRP3 inflammasome, proinflammatory factor levels and pyroptosis in the hearts of DCM mice.

We further investigated the mechanisms by which ICA may ameliorates NLRP3 inflammasome and pyroptosis in diabetic cardiomyocytes. ICA exerts its protective effect in diabetes complication through different signaling pathways and can exert anti-fibrotic effect with its regulation of Apelin/SIRT3, p65/c-Jun, and TGF-β1/Smad signaling (Ni et al., 2020; Zhang et al., 2021; Li et al., 2013). In addition,ICA improve diabetic vascular endothelial dysfunction include anti-ER stress, promotion of angiogenesis, anti-autophagy, and regulation of vascular systolic and diastolic function (Yao et al., 2021; Tang et al., 2015). Importantly,ICA has been regarded as a potential agonist of SIRT3 (Zeng et al., 2019). SIRT3, an NAD^+^ dependent mitochondrial deacetylase, positively modulates many cellular processes, including energy metabolism, mitochondrial biogenesis, protection against oxidative stress and inflammation, maintain mitochondrial homeostasis (Zhou et al., 2022; Wang et al., 2023; Palomer et al., 2020). In this article, we demonstrate that SIRT3 protein expression was reduced, but administration of ICA was associated with enhanced SIRT3 expression. It has been found that SIRT3 deficiency aggravated hyperglycemia-induced mitochondrial damage, increased ROS accumulation, promoted necroptosis, possibly activated the NLRP3 inflammasome, and exacerbated DCM in the mice (Song et al., 2021). Induction of SIRT3 expression reversed diabetes-induced hyperacetylation and functional impairments of mitochondrial enzymes (Jin et al., 2022). Interestingly, we revealed that a SIRT3 inhibitor markedly attenuated the inhibitory effects of ICA on NLRP3 inflammasome activation and pyroptosis in DCM mice, suggesting that ICA may exert its protective effects in a potentially SIRT3-dependent manner.

Previous data also shows that SIRT3 regulates the acetylation of TFAM and preserves the mitochondrial function in diabetic rat heart. Reduced SIRT-3 expression decreased mtDNA binding activity of TFAM and reduced transcription of mtDNA encoded genes (Bagul et al., 2018). TFAM downregulation promotes mtDNA release into the cytosol, induces cytosolic mtDNA stress, subsequently activates the cGAS-STING signaling pathway (Li et al., 2022). Therefore, we further confirmed that ICA was associated with reduced mitochondrial oxidative stress, enhanced TFAM deacetylation, and decreased cytosolic mtDNA levels. However, inhibition of SIRT3 markedly attenuated the myocardial protective effects of ICA. In summary, these findings suggest that ICA may attenuate inflammation and pyroptosis via a potential SIRT3- TFAM-mtDNA-NLRP3 signaling pathway in DCM mice heart.

Some questions remain unanswered due to the limitations of our study. Firstly, we did not detect any mtDNA leakage channels in the mitochondria of diabetic cardiomyocytes, nor did we establish whether inhibiting mtDNA by DNase I could prevent NLRP3 inflammasome activation. Secondly, the results showed that ICA could inhibit mtROS production in diabetic cardiomyocytes, but the precise mechanism was not explored in depth. Additionally, the proposed SIRT3-TFAM-mtDNA-NLRP3 signaling cascade is relatively long, and our study mainly provides correlative evidence rather than sufficient mechanistic validation. Further in-depth experiments are required to fully verify the sequential regulatory relationships among these molecules.

In conclusion, our study suggests that the escape of mtDNA into the cytosol caused by TFAM acetylation may trigger the initiation of NLRP3 inflammasome-dependent pyroptosis and the proinflammatory response, while ICA may decreased cytosolic mtDNA levels by regulating SIRT3-mediated TFAM deacetylation, thereby potentially inhibiting the inflammation and pyroptosis in the hearts of DCM mice.

## Data Availability

The original contributions presented in the study are included in the article/supplementary material, further inquiries can be directed to the corresponding authors.
